# Intravesical gemcitabine and docetaxel vs. re‐induction Bacillus Calmette Guerin as first‐line salvage therapy for non‐muscle invasive bladder cancer

**DOI:** 10.1002/bco2.70012

**Published:** 2025-04-03

**Authors:** Kylie Yen‐Yi Lim, Tran Ngoc An Huynh, Gavin Wei, Jincy Kuriakose, Obaidullah Fazli, David Pook, Sarah Ransley, Janice Downie, Scott Donnellan, Weranja Ranasinghe

**Affiliations:** ^1^ Department of Urology Monash Health, Casey VIC Australia; ^2^ School of Clinical Sciences Monash University Clayton VIC Australia; ^3^ Department of Pharmacy Monash Health Clayton VIC Australia; ^4^ Department of Oncology Monash Health Clayton VIC Australia; ^5^ Department of Anatomy and Developmental Biology Monash University Clayton VIC Australia

**Keywords:** BCG failure, gemcitabine‐docetaxel, non‐muscle invasive bladder cancer, salvage therapy

## Abstract

**Objectives:**

To compare the outcomes between re‐induction Bacillus Calmette‐Guérin (BCG) and sequential intravesical gemcitabine‐docetaxel (Gem/Doce) therapy in patients with high‐grade (HG) non‐muscle invasive bladder cancer (NMIBC) following failure of initial induction BCG.

**Materials and methods:**

We retrospectively identified patients who received induction BCG therapy between 2017 and 2023. Inclusion criteria were high‐grade NMIBC recurrence post‐BCG induction, with subsequent treatment by either re‐induction BCG or Gem/Doce.

**Results:**

From 2017 to 2023, 140 patients received induction BCG, with 30 (21.4%) showing persistent HG NMIBC. Of these, five (16.7%) were treated with re‐induction BCG and 11 (36.7%) with Gem/Doce. In the re‐induction BCG group, four patients (80%) had HGTa and one (20%) had HGT1. In the Gem/Doce group, eight patients (73%) had HGTa, two (18%) had HGT1 and one (9%) had carcinoma in situ (CIS). Initial post‐treatment cystoscopy showed recurrence in one re‐induction BCG patient (20%) with HGT1 and CIS and in two Gem/Doce patients (18%) with HGTa. No adverse events were reported with Gem/Doce.

**Conclusion:**

Our initial experience with intravesical Gem/Doce suggests that it is better tolerated, with fewer adverse events and comparable recurrence rates at three months, compared to re‐induction BCG in patients with BCG‐failure NMIBC.

**Abbreviations**
AE: Adverse events
BCG: Bacillus Calmette‐Guérin
CIS: Carcinoma‐in‐situ
Gem/Doce: Gemcitabine‐docetaxel
HG: High grade
NMIBC: non‐muscle invasive bladder cancer
RFS: Recurrence‐free survival

Bladder cancer is the 11th most common cancer in Australia, with non‐muscle‐invasive bladder cancer (NMIBC) accounting for approximately 75% of diagnoses. International guidelines from the American Urological Association and the European Association of Urology recommend patients with NMIBC be treated with transurethral resection of bladder tumour, followed by intravesical bacillus Calmette‐Guérin (BCG) therapy.^1^, ^2^ However, 20–40% of patients will have recurrent or persistent disease following BCG therapy, classified as BCG failure.^3^ While radical cystectomy (RC) remains the standard treatment for these patients, some may be poor surgical candidates or opt for bladder preservation. Consequently, many clinicians use re‐induction BCG, but this has historically shown low efficacy, with a recurrence‐free survival rate (RFS) of 35%.^4^

The ongoing global BCG shortage has further emphasised the need for alternative bladder‐preserving options. One promising alternative is the sequential administration of intravesical gemcitabine‐docetaxel (Gem/Doce), which has shown a two‐year high‐grade (HG) RFS of 51.4%.^5^ Despite being based on retrospective studies, Gem/Doce has received mentions as an alternative treatment in major international guidelines.^1^, ^2^

We introduced Gem/Doce therapy at our institution in 2021, becoming the first Australian centre to develop a routine program for this treatment. Our aim was to compare the efficacy of intravesical Gem/Doce and re‐induction BCG for patients with high‐grade (HG) NMIBC recurrence following BCG therapy.

We retrospectively identified patients who received induction BCG therapy at a multi‐site tertiary institution in Victoria, Australia, between 2017 and 2023. Inclusion criteria were patients with HG recurrence after induction BCG, who received either re‐induction BCG or Gem/Doce. Data recorded included patient demographics, comorbidities, bladder cancer history and treatment details, side effects and outcomes. Side effect symptoms were collected at each intravesical therapy visit and graded according to the CTCAE version 5.0 (National Cancer Institute: Common Terminology Criteria for Adverse Events [CTCAE] version 5.0).

The BCG protocol included induction with 81 mg of BCG weekly for six weeks. Re‐induction BCG is a repeated course of induction BCG. Gem/Doce, introduced in November 2021, also involved weekly treatment for six weeks. Patients received 1000 mg gemcitabine, followed by 37.5 mg of docetaxel, instilled via a closed system transfer device. Surveillance post‐reduction BCG or Gem/Doce involves cystoscopic evaluation under anaesthesia 6–8 weeks from the last treatment dose. At our institution, standard white‐light cystoscopy was used for surveillance. Random bladder biopsies and directed biopsies of suspicious lesions were performed to confirm recurrence or persistent disease.

At our institution, a BCG maintenance course consisted of 81 mg of BCG, administered weekly for three weeks and repeated every three to six months for one to two years. Maintenance therapy for Gemcitabine (1000 mg) and Docetaxel (37.5 mg) was given every three months for two years, with regular cystoscopic and cytologic evaluation.

Descriptive statistics were performed. This study was approved by the local ethics committee QA/87943/MonH‐2022–321 914(v1).

During our study period, 140 patients underwent induction BCG for NMIBC. Thirty (21.4%) patients had HG non‐muscle invasive persistence following induction BCG. Of these, five (16.7%) were treated with re‐induction BCG, eleven (36.7%) received intravesical Gem/Doce and others received alternative therapies. Out of those who had re‐induction BCG, four (80%) patients had HGTa and one (20%) HGT1 prior. Out of those who had Gem/Doce treatment, eight (73%) patients had HGTa, two (18%) patients had HGT1 and one patient had just CIS (9%) prior.

Patients who received re‐induction BCG and Gem/Doce were predominantly male with a median age of 74 and 76 years old respectively. The median follow‐up duration was 28.14 months for the re‐induction BCG group and 8.72 months for the Gem/Doce group. This difference is attributed to the introduction of Gem/Doce as a treatment option in November 2021.

On initial post‐treatment cystoscopy, one patient (20%) from the re‐induction BCG group had HGT1 with CIS recurrence, and two patients (18%) from the Gem/Doce group had HGTa recurrence. Two patients from the re‐induction BCG developed metastatic disease. One patient from the Gem/Doce group developed HG upper tract UC in the kidney with distant metastases. Time to metastases for the patient who received Gem/Doce was 8.6 months compared to a median of 14.1 months in the re‐induction group.

Both groups of patients completed all six doses of treatment. While both groups exhibited similar symptom profiles, side effects were more frequent and varied in the re‐induction BCG group. In the re‐induction BCG group, the most commonly reported symptoms were dysuria (80%), urgency (60%), frequency (60%), haematuria (40%), lethargy (40%), fever (20%) and bladder spasms (20%). In contrast, the Gem/Doce group reported lower overall rates of adverse events, with patients commonly reporting urgency (36%), frequency (36%), dysuria (21%), lethargy (18%) and bladder spasms (18%). In both groups, the symptoms were mild (CTCAE grade 1–2) and self‐limiting, with no patients requiring hospitalisation post‐treatment.

BCG‐recurrent NMIBC remains a challenging condition. While RC is the standard treatment, it is not suitable for all patients. Re‐induction BCG is a possible option but has poor efficacy with difficult accessibility. Gem/Doce provides an alternative bladder‐preserving option. Currently, our response rates align with the complete response rate at first surveillance of 74% as reported by Cheveru et al.^6^ A recent retrospective analysis with a median follow‐up of 49 months, reported longer‐term outcomes with a 30% HG RFS and a 91% 5‐year cancer‐specific survival rate in patients treated with Gem/Doce after BCG failure.^6^ Despite evidence for Gem/Doce for salvage therapy emerging in this past decade and a systematic review published this year identifying only two studies, the data is demonstrated to be highly promising.^5^

Our initial experience with Gem/Doce demonstrates that it is well‐tolerated, with local symptoms reported as mild and self‐resolved. A recent retrospective study looking at treatment naïve NMIBC found that BCG patients were more likely to discontinue treatment than Gem/Doce patients because of the side effects (9.2% vs. 2.9%, p = 0.02).^7^

As the first centre in Australia to initiate a routine, specific program for doublet therapy, we found it feasible and relatively easy to institute Gem/Doce. To facilitate the introduction of this double therapy, local institution approval was obtained in partnership with the pharmacy department. An institutional protocol was established based on other international protocols. Gem/Doce was already readily available at our hospital for treatment of other non‐urological malignancies, making it an accessible medication. From a practical standpoint, the hurdles encountered were sourcing the closed system transfer device and providing comprehensive training to staff administering the therapy. As this treatment option is now well‐established in our institution, subsequent treatments have been conducted with ease.

Additional benefits of Gem/Doce include its cost‐effectiveness compared to BCG therapy.^8^ Unlike BCG, Gem/Doce is not affected by recent production shortages, ensuring more consistent availability. As a chemotherapy combination also used in the treatment of non‐urological malignancies, it is widely accessible. Another significant advantage of Gem/Doce is its stable shelf life after preparation, allowing for advanced preparation and facilitating administration in remote areas without biohazard facilities or closed‐access systems required for BCG preparation.

In contrast, reconstituted BCG must be stored at 2–8°C, protected from light. While earlier recommendations suggested an in‐use stability of only 2 hours, recent data indicate that BCG may remain stable for up to 72 hours under appropriate conditions.^9^, ^10^ However, logistical challenges related to storage, handling and unpredictable supply continue to impact its use. Given these factors, Gem/Doce represents a feasible, bladder‐sparing, affordable and effective alternative, particularly in lower‐income countries.

The limitations of this study include the retrospective nature, small sample size and short follow‐up duration. Patient symptoms were also self‐reported symptoms, rather than assessed using a standardised AE assessment. Furthermore, patients who received alternative treatments were excluded from the cohort, which limits the generalisability of the findings.

Our initial experience with intravesical Gem/Doce demonstrates that it appears to be safe and better tolerated compared to re‐induction BCG, with fewer reported adverse events. Both therapies showed comparable recurrence rates at the 3‐month surveillance cystoscopy. These findings suggest that Gem/Doce may serve as a well‐tolerated, bladder‐preserving alternative for patients with BCG failure NMIBC. Further multi‐institutional, randomised studies with long‐term follow‐up are needed to validate these findings.

## CONFLICT OF INTEREST STATEMENT

There is no conflict of interest associated with this publication and no financial support was required at time of submission.
